# Underutilized Green Banana (*Musa acuminata AAA*) Flours to Develop Fiber Enriched Frankfurter-Type Sausages

**DOI:** 10.3390/foods10051142

**Published:** 2021-05-20

**Authors:** Diego Salazar, Mirari Arancibia, Lenin Calderón, María Elvira López-Caballero, María Pilar Montero

**Affiliations:** 1 Facultad de Ciencia e Ingeniería en Alimentos, Universidad Técnica de Ambato, Av. Los Chasquis y Rio Payamino, Ambato 180206, Ecuador; marancibias@uta.edu.ec (M.A.); lcalderon7163@uta.edu.ec (L.C.); 2 Facultad de Veterinaria, Universidad Complutense de Madrid, 28040 Madrid, Spain; 3 Instituto de Ciencia y Tecnología de Alimentos y Nutrición (ICTAN-CSIC), Calle José Antonio Novais 10, 28040 Madrid, Spain; mpmontero@ictan.csic.es

**Keywords:** green banana flours, fiber, fat-replaced, Frankfurter-type sausage, chilled storage, wheat-free

## Abstract

This study aimed to develop a fiber-enriched Frankfurter-type sausage by incorporating underutilized green banana flours as a meat extender, replacing wheat flour with banana flours (8%). A low-fat formulation substituting 12% pork fat with 24% banana peel flour was also studied. Sausages were stored at 4 °C/15 days. Cooking loss was low (5.6–4.1%) in all formulations and the substitution of wheat flour with banana flour did not modify moisture and protein composition, while carbohydrate, fiber, and ashes varied with the flour composition. In the low-fat sausages, fiber carbohydrate and ashes increased the most. Texture and color parameters were very similar for high-fat sausages throughout storage, although low-fat sausage showed higher hardness, while chewiness, L*, and whiteness tended to decrease. During the first week of storage, the microbial growth was scarce and then, an increase, except in the low-fat batch, in which growth remained constant. *Enterobacteria* and *Staphylococcus aureus* were not detected during storage. Sensory attributes throughout storage were very similar for all high-fat sausages; the odor in the formulations was defined as “different” but not unpleasant. The low-fat sausages, defined as a new product different from conventional sausages, were well accepted by the panelist. Banana flours are a suitable ingredient option to add nutritional value to Frankfurter-type sausages, which can be consumed by the wheat allergic population.

## 1. Introduction

Recently, the meat industry has focused on developing healthier products due to consumer demands for high nutritional value products with balanced formulations. One of the strategies followed by the industry is foodstuff reformulation, adding ingredients that improve health and limiting undesirable components [1,2,3]. In the meat industry, different sausage types, such as Frankfurter, Vienna, Bratwurst, and Ranchera, among others, are examples of widely consumed cooked gel-emulsion sausages that are losing market on account of their reduced health components. Likewise, advertising that regular consumption of meat and meat products is associated with a series of diseases such as colon cancer, cardiovascular disease, and obesity contributes to an increase in the number of detractors towards the consumption of this type of foods [4,5,6]. In addition, there is a large sector of the population, especially in the wealthiest countries, which frequently consume meat and meat products, and have a poorly balanced diet [7,8]. Hence, there is a double interest, from the industry and from the consumer in the need to develop healthier meat products.

There are different possibilities to achieve healthier meat products, including the addition of ingredients such as fibers or resistant starch as binders that would substantially improve the product [9,10]. In this sense, several scientific studies have developed sausages in which fibers and starches are incorporated to replace animal fat, becoming one of the most effective strategies in meat-product innovations. Fiber-enriched meat products (≤2–3%) are generally limited by sensory acceptability [3,9,10]. Starches, especially modified ones, are widely used in the industry for their binding and texturizer properties and are also suitable as extenders. However, they would not have the healthy aspects that fibers and other nutrients could confer [11], although more fiber is required to be used as a fat substitute. Thus, it is important to consider that with the inclusion of some ingredients, sensory properties may be very different from those of the original product and may not be suitable for consumer taste [11,12,13] and their incorporation into mixtures is required [1]. Therefore, a compromise between the organoleptic quality and the quantity and type of ingredient to be included as a substitute for the most undesirable ingredients must be sought [14]. The industry is relentlessly searching for ingredients to improve the health characteristics of meat products and at the same time, make them economically feasible. Fibers are not an economical component and the formulation sometimes becomes nonviable. However, the inclusion of starches from different origins is a much more cost-effective alternative to produce sausages with techno-functional and nutritional benefits [1,3,9].

The banana crop is one of the main resources in the economy of several South American countries, like Ecuador, being the banana world production ~113 million tons [15]. Banana is usually exported; however, around 25% of the total production is rejected, mainly because it does not meet class “A” quality requirements: variety, number and size of fingers per hand, color, appearance, caliber, packaging, and phytosanitary conditions [16,17], although that does not mean it should not be consumed [18]. In this sense, banana flours stand out as one of the best options considering their health and nutritional properties [8,19], and their low cost. Researchers and the food industry have been interested in banana flours for their potential techno-functional properties. The meat industry could offer organoleptically acceptable products if functional additives with exceptional nutritional value were to be incorporated. Banana flours are a low-cost ingredient that, instead of being discarded, can be used as an alternative to traditional flours and starches [12,20,21]. In this connection, green banana is characterized by being rich in resistant starch and fiber, phenolic compounds, tannins, and certain minerals as phosphorous, magnesium, copper, and iron [8,19]. Resistant starch is associated with promoting beneficial physiological effects that include blood glucose attenuation and improvement of the gastrointestinal system, thus reducing the food glycemic index [22]. For all the aforementioned reasons, the use of green banana flours could be of great interest as a functional ingredient.

The present study aims to evaluate the effect of the inclusion of different green banana flours from the pulp, peel, or the whole banana, on the technological, physicochemical, microbiological, and sensory properties of cooked gel-emulsion sausages/Frankfurter-type sausages. Besides, the use of banana peel flour as a fat replacer in the formulation of sausages will be evaluated. The evolution of the sausages during 15 days of chilled storage was also studied.

## 2. Materials and Methods

### 2.1. Elaboration of Banana Flour

The green bananas (*Musa acuminata AAA*) were purchased in a local market of Ambato (Ecuador). The fruits were selected according to homogeneous green color and uniformity. The bananas were thoroughly washed in water, and after that, they undergo different processes depending on the flour to be obtained: bananas were cut into slices ~0.03 cm thick (whole banana) or skinned and cut into slices ~0.03 cm thick (pulp). The peel resulting from the process to obtain the pulp was cut with a length of ~0.5 cm (peel). All batches were uniformly spread out in trays to be dried in a convective dryer (Gander MTN) at 60 °C until reaching the constant moisture (around 12%). Later, all the dried material of each batch was crushed in an industrial mill (Inox Equip, Riobamba, Ecuador), and the flours obtained were: whole banana flour, banana pulp flour, and banana peel flour.

The composition of flours was: whole banana flour (moisture 11.32%, fat 0.87%, protein 3.53%, ash 3.76%, fiber 3.51%), banana pulp flour (moisture 9.96%, fat 0.15%, protein 3.41%, ash 2.64% fiber 1.28%), peel banana flour (moisture 9.64%, fat 3.51%, protein 4.24%, ash 6.35%, fiber 10.26%). Wheat flour (moisture 14%, fat 2%, protein 12%, ash 0.65%, fiber 2.75%) was used for control purposes.

### 2.2. Frankfurter-Type Sausage Preparations

Frankfurter-type sausages were produced according to a standard procedure, where the control formulation (Control) is composed of meat protein (31% beef and 27% pork), 15% pork-back fat, 9% of ice, 8% of wheat flour, and 6% of seasoning (1.6% sodium chloride, 200 mg/kg sodium nitrite, 0.05% polyphosphates and 0.05% ascorbic acid powder, garlic powder, onion powder, pepper, nutmeg, cinnamon, and sugar). Three types of new formulations were made changing the wheat flour in the same percentage (8%) by whole banana flour (HF-WBF), banana pulp flour (HF-PUBF), or banana peel flour (HF-PEBF); the rest of the ingredients were the same in all batches (these sausages containing the same fat as the control are named as “high fat, HF). A low-fat, fiber-enriched formulation was also elaborated, reducing the incorporation of pork back fat at 3% (instead 15%) and substituting the 8% of wheat flour by 24% of banana peel flour to obtain a product of potential nutritional interest with respect to its fat and fiber content (low-fat fiber-enriched sausages); the other ingredients were not modified, constituting the batch LF-PEBF.

For sausages production, meats were ground in a mincer (Mainca PM-21 Spain) for all batches. Then the minced meat, fat, and 25% of crushed ice were homogenized in a cutter (Mainca CM-21 Spain) for 2 min, and subsequently, the seasoning and 25% of crushed ice were added and homogenized 2 min; finally, each flour and 50% crushed ice was added in the corresponding formulas, homogenizing 1 min. Each final batter was stuffed into an artificial Viscofan casing (16 mm diameter/150 mm length). Later, sausages were cooked in a water bath at 80–85 °C until the core of the product reaches 73 °C (~20 min). After heating, the sausages were cooled in a cold bath until an internal temperature of 30 °C. Finally, sausages were packed in high-density polyethylene plastic food bags and refrigerated at 4 °C for 15 days. The elaboration of the sausages was conducted in triplicate (three independent experiments).

### 2.3. Cooking Loss

The yield loss in processing was determined during heat treatment; for that, the Frankfurter-type sausages were cooked (amounts of approximately 1 kg) and weighed before and after cooking. Cooking loss, expressed as a percentage, was evaluated as the initial weight before cooking minus the final weight after cooking. The results are expressed at least by triplicate.

### 2.4. Proximal Analysis

Proximate composition (moisture, ash, protein, and fat) was evaluated following the official methods AOAC 19 927.05, AOAC 923.03, AOAC 2001.11, and AOAC 2033.06, respectively (AOAC, 2005) [23]. Carbohydrates were estimated by the difference in moisture, protein, ash, fat, and fiber content. The protein content was calculated by Nitrogen determination using the factor of 6.25. The determination of fiber was carried out using the enzymatic-gravimetric method (AOAC 985.29) (PRT-701.03-019, 2011) (AOAC, 2005) [23]. All determinations were performed in triplicate and the results were expressed as a percentage.

### 2.5. Energy Value

Calorie content was estimated at ×100 g, as the overall sum of calories of the individual components is the energy value for each component: fat (×9 kcal/g), protein (×4 kcal/g), carbohydrate (×4 kcal/g), and fiber (×2 kcal/g).

### 2.6. pH and Acidity

The pH of samples was measured at room temperature on homogenates of sausages in water (1:10 *w*/*v*), using a digital pH-meter (HANNA HI 9126, Rhode Island, RI, USA). The acidity was determined by titration with NaOH 0.1 N, using phenolphthalein as an indicator according to the methodology described in AOAC, (2005) and it was expressed as g/100 g of lactic acid. All tests were carried out in triplicate.

### 2.7. Texture

The texture profile analysis (TPA) was performed in a texturometer (CT3 Brookfield, Scarsdale, NY, USA). Once removed the casing of the sausages, each batch was cut into cubes (1.5 cm wide, 1.5 cm long, and 1.0 cm high). A double compression was made up to 75% deformation (normal stress) with five seconds waiting time between compressions. A head speed of 1 mm/s and 25 kg load cell was used. The parameters to be measured were hardness—peak maximum force on the first compression (N), cohesiveness—the ratio of positive areas in the two compression cycles and represents the work required to compress the food a second time compared to what has been necessary to compress it the first time (dimensionless), springiness—the height that the food recovers after the first compression (mm), adhesiveness—the negative force area obtained after the first compression, which represents the work required to separate the plunger compression food (N × mm) and chewiness—the product of hardness × cohesiveness × springiness (N × mm). Analyses were performed at 1, 3, 6, 9, 12, and 15 days of chilled storage.

### 2.8. Color Determination

Color CIE Lab tristimulus parameters, L* (lightness), a* (red/green) b* (yellow/blue), of cross-sections of sausages, were determined with a Hunter Lab Colorimeter (mini Scan 4500 L EZ, Hunter Associates Laboratory INC, Reston, VA, USA) calibrated with an illuminator D65 (natural light) and standard observer D10. The color was expressed by the parameters L *, a *, and b *. In addition, the chroma polar coordinate or saturation C* was calculated from the expression C* = √ (a* 2 + b* 2) and Hue (h°) = arctang (b*/a*) to a* and b* positives. Furthermore, it was determined the whiteness index, according the equation W = 100 − [(100 − L*)2 + (a*2 + b*2)]1/2. For the calibration of the colorimeter, before measurements were done, the white tile standard was used. At least 15 measurements were performed in different areas of the samples. Analyses were performed at 1, 3, 6, 9, 12, and 15 days of chilled storage.

### 2.9. Microbiological Analysis

Ten grams of Frankfurter-type sausages were aseptically placed into a sterile stomacher bag. They were then homogenized with 90 mL of buffered 0.1% peptone water (Difco, Le Pont de Claix, France) in a stomacher homogenizer (Model 400 C, Seward, London, UK) for 1 min at room temperature. For each sample, appropriate serial decimal dilutions were prepared in peptone water. Mesophilic aerobic bacteria were determined in pour plates of PCA agar (Difco) incubated at 30 °C for 72 h; Mold and yeast were spread plated on Rose Bengal Agar (RBC) (Difco) and incubated at 25 °C; *Enterobacteriaceae* on a double layer of Violet Red Bile Glucose Agar (VRBG) (Acumedia, MI, USA) incubated at 30 °C/72 h; and *Staphylococcus aureus* on Baird Parker agar (Difco) supplemented with egg yolk tellurite were incubated at 30 °C for 48 h. All analyses were performed in triplicate. The test was carried out on day 1, 4, 8, 12, and 15 days of chilled storage.

### 2.10. Sensory Analysis

Fifteen semi-trained panelists assessed attributes such as smell, taste, and overall acceptability of the sausages. For this, the panel received training in previous sessions. A sensory acceptance test was performed using a 5-point hedonic scale (5—liked very much; 4—like moderately; 3—neither liked nor disliked; 2—disliked moderately; 1—disliked very much). Two cylinders (1.6 cm in diameter × 3 cm length) of grilled samples without casing were immediately given to the panelist for evaluation. A glass of water and salted crackers for a palate-cleansing were also provided. Analyses were carried out on day 1, 3, 6, 9 12, and 15 days of chilled storage.

### 2.11. Experimental Analysis

The data were processed and analyzed with the GraphPad Prism 5.0 program (GraphPad Software, San Diego, CA, USA). Analysis of variance was performed using the one-way or two-way ANOVA test, and when this was significant, the comparison of the means was performed using the Tukey test (α = 0.05).

## 3. Results and Discussion

### 3.1. Cooking Loss

The cooking loss was different in all batches (*p* < 0.05) even though none differ more than 1% from the Control (Table 1). This relatively low cooking loss (around 5%) could be due to the high flour content, 8% in high-fat formulations of cooked gel-emulsion Frankfurter-type sausages, and 24% in the low-fat formulation. The high cooking yield (<10% cooking loss) is indicative of the good quality of meat products since it evidences a high water holding capacity during cooking [24]. Accordingly, beef sausages including commercial pineapple dietary fibers slightly reduced the cooking loss but did not differ from the values obtained by the Control sausages (without pineapple fibers) [25]. Pereira and Maraschin [18] observed a slightly lower cooking loss (between 2.20 and 4.39%) in sausages made by substituting 50% pork fat with emulsions of sunflower oil with water and banana flour (ratio 1:2:2) in different proportions, showing an increase in cooking loss with the replacement of fat. Choe et al. [24] in Frankfurters-type sausages in which fat was replaced with a mixture of pigskin and wheat flour, reported a cooking loss of about 4%. Other authors have observed a more significant cooking loss in different meat products with the addition of flours or fibers. Thus, Alves et al. [1] observed a cooking loss of 14–10% in Bologna-type sausages reduced in fat by substituting it for a gel made with pork skin, whole banana flour, and water (ratio 1:2:2) at various proportions (between 20 and 60%). The lowest amount of flour added was 7.65%, and the cooking loss of sausages with these formulations was around 10%. The addition of fiber in the formulations improves the cooking yield [24,26].

### 3.2. Proximal Composition and Nutritional Estimations

The proximal composition of the different sausage formulations is shown in Table 1. The formulas that contained whole banana flour (HF-WBF) presented the same water content as the Control and were slightly lower in those that contained pulp flour (HF-PUBF) (*p* < 0.05). Frankfurter-type sausages are considered to be a high-intermediate moisture food, with values oscillating between 50–75% [27]. In the present study, all batches were within this range. According to the results, replacing wheat flour with banana peel flour (LF-PEBF) contributes to a higher water content, which may be attributable to its significantly higher fiber content. Rosero-Chasoy and Serna-Cock [8], studying sausages formulated with banana peel flour, suggested that a higher water content may be favored by the greater content of certain hydrophilic amino acids in the composition of the flour, such as valine, methionine, threonine, and cysteine. However, there is a slight decrease in water content when the addition of banana peel flour is accompanied by a marked decrease in fat (12%). A decrease in water content was observed in chicken sausages as oat bran content in the formula increased [28].

The ash content was different in most of the sausages (*p* < 0.05) (Table 1); these differences are attributable to the amount of ash of each type of flour and the concentration at which they were included in the formulations. Banana peel flours have a greater ash content (~6%) and consequently, HF-PEPF sausages contained a higher amount of ash, especially LF-PEBF, as these sausages had a higher percentage of flour in the formulation. The ash content of the sausages in this study was lower than those presented by Alves et al. [1] (~3–4%) in a Bologna low-fat sausage in which pork fat was replaced with a gel formulated with pork skin, banana flour, and water (ratio 1:2:2).

Despite the significant differences in protein content in all batches, the values did not differ by more than 0.59% compared with the control sausage (Table 1).

These results were similar to those presented by Choe and Kim [29] in sausages with a wheat fiber mixture as a fat replacer and Alvarado-Ramírez, et al. [30] in sausages with carrot powder as an ingredient, with 12.60% and 12.85% of protein, respectively.

The substitution of wheat flour (Control) with banana flours (HF-PUBF, HF-WBF, and HF-PEBF) at equal proportions showed a very slight decrease (*p* < 0.05) in the fat content of the sausages (Table 1). These minor variations could be due to the different fat content of the flours or to a slight fat loss by cooking. As expected, the low-fat sausages (LF-PEBF) contained around half of the fat of the Control (*p* < 0.05). Some authors also observed cooking losses, mainly of fat, when the formulation contained fibers as partial fat substitutes [13,29,30,31] as fibers have a low capacity to retain and/or absorb fat [32].

The fiber content of the sausages depends directly on the composition and concentration of flours used in the formulation (*p* < 0.05) (Table 1). Thus, a greater amount of fiber in the flour favors a higher fiber content in the sausages, as shown in the case of HF-PEBF (*p* < 0.05) as compared with the rest of the formulas with the same amount of flour (Control, HF-PUBF, and HF-WBF). In agreement with nutritional claims, the high-fat sausage formulations could be considered as a “source of fiber” since all of them have more than 3% fiber (3 g fiber/100 g of product). Moreover, sausages including banana peel flour, both high and low in fat (HF-PEBF and LF-PEBF, respectively), could be labeled as “high fiber” products because both of them contain more than 6% fiber [33]. In meat formulations, the fiber content is often notably lower than the above-mentioned percentage to avoid adverse sensory effects, as it has been previously reported when including citrus fiber (0.5–2%) [34] or banana flour (0.8–4.8%) [1] in Bologna-type sausage formulations or dietary fiber from makgeolli lees (0–2%) in reduced-fat Frankfurters [26].

The highest content of non-fiber carbohydrates was observed in HF-PUBF and the lowest in HF-PEBF sausages, while HF-WBF and the Control showed similar and intermediate values (*p* < 0.05) (Table 1). These results are directly proportional to the number of carbohydrates in the flours used to formulate the sausages except for the Control sausages (wheat flour has fewer carbohydrates than whole banana flour). Similarly, the carbohydrate content in the low-fat sausages (LF-PEBF) is quite high due to the elevated content of flour in this formulation.

Concerning the caloric content of sausages (Table 1), high-fat samples had similar values (*p* < 0.01); HF-WBF and HF-PUBF registered a lower caloric content compared to the Control (2 and 5%, respectively). However, despite having the same proportion of flour, HF-PEBF showed a lower caloric content (11%) probably due to the lower amount of carbohydrates and fat of this formulation. In addition, the low-fat, high-fiber sausages contained 24% less energy (Table 1). A decrease in the caloric content was obtained in sausages when fat was replaced with olive oil, flax, or konjac gels (up to 165 kcal/100 g product) [35], or with makgeolli lees fiber (about 139.30 kcal/100 g product) [26]. Considering the recommendations for a daily balanced diet, the World Health Organization [36] recommends that the energy content should be composed of a variable contribution of 55–57% carbohydrates, 15–30% fat, and 10–15% protein. In products such as Frankfurter-type sausages, the proportion is very far from the desirable energy balance, despite the improvement in the sausage formulations with the incorporation of green banana flours and the reduction of fat, as in the LF-PEBF batch. On the other hand, it is not necessary to reach this balance in each daily consumed product, but it is desirable in relation to the total daily food intake; in this sense, there is no doubt that for some consumers, meat products constitute an important source of energy in the diet, so achieving a formulation with a more balanced energy content in meat products is a desirable challenge. In the LF-PEBF sausages, there has been a change in trend with the decrease of the energy content from fat (to 78% in Control up to 52% in LF-PEBF) and the increase of that from carbohydrates (from 7.75% in Control up to 29% in LF-PEBF sausages), while the protein-energy intake is in line with the suggested balance.

### 3.3. pH and Acidity

pH variations between sausages were very small (*p* < 0.01) (Table 1). These slight variations may be due to the different pH of each flour; banana peel flour has a pH close to 4.7, banana pulp flour 5.7, while the pH of wheat flour ranges between 6.2 to 6.5 [37,38]. This fact could explain the downward trend in pH obtained in the sausages with banana peel flour regardless of the fat content in the formulation. The fibers interact through electrostatic association (attraction and repulsion) between protein polar and non-polar groups, resulting in pH variations in Frankfurters with inulin and pectin [31]. Similar results were obtained in chicken sausages enriched with makgeolli lees fiber, with pH values between 6.4 and 6.5 [39], and in a Bologna-type sausage with pea fiber, with a pH range of 6.47 to 6.63 [40]. In both studies, it was observed that the pH of meat products is affected by the presence of dietary fiber. According to Araya-Quesada et al. [41], the fiber and banana starch plays a neutralizing role in the sausage, which prevents the decrease or increase of pH in each sample during storage time, making sure that the sausages do not undergo sensory and/or texture changes consequence of pH variations. On the other hand, in the present work, all lots presented a pH close to neutrality, probably favored by the alkalinization process caused by endogenous changes, such as the release of products as threonine, serine, and methionine, which are products of the degradation of protein chains in the sausages during heat treatment [42].

The acidity values were low and quite similar for all formulations, with the highest values obtained in HF-PUBF and LF-PEBF (*p* < 0.05) (Table 1). According to Mehta et al. [32], the slightly acidic variation found in the different sausages could be due to the presence of organic acids in the flours.

### 3.4. Textural Properties

**Figure 1:**
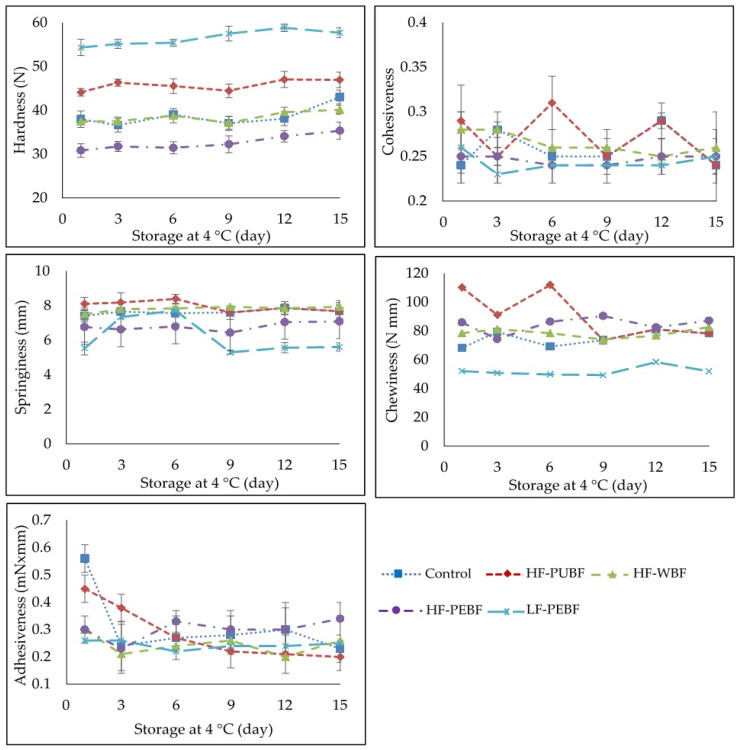
Textural profile analysis of Frankfurter-type sausages formulated with green banana flours: Control (High fat and wheat flour), HF-PUBF (High fat and banana pulp flour), HF-WBF (High fat and whole banana flour), HF-PEBF (High fat and banana peel flour), LF-PEBF (Low-fat and banana peel flour).

The replacement of wheat flour with banana flours in the high-fat formulation led to slight modifications in sausage hardness (*p* < 0.05). Differences among lots depended on the type of flour used in the formulations. Compared to the Control, hardness was higher for HF-PUBF and lower for HF-PEBF, while the values were the same for HF-WBF (Figure 1). Moreover, a noticeable increase in hardness was obtained in the low-fat, enriched fiber sausages (LF-PEBF) (*p* < 0.05), attributed to the high fiber content as reported by Atashkar et al. [43]. This behavior was consistent throughout the storage period. Tahmasebi et al. [44] reported that in meat sausages formulated with different flours (pigeon-pea flour and cornflour), sunflower oil and replacements (walnut paste, sesame paste, or a mixture of the two), all of them in different concentrations, the increment of pigeon-pea flour resulted in sausages with increased hardness because of the higher content of protein and fiber of this flour (compared to corn flour), resulting in a stronger gel, more resistant to compression. Cohesiveness was very similar and stable in all formulations, as was springiness, except in LF-PEBF, in which values were slightly lower (*p* < 0.05) on the first day of storage, increasing variability throughout chilled storage and reaching similar values to the initial one after 9 days. In beef emulsion modeling systems prepared with tropical flours (breadfruit or banana) at different inclusion levels (0, 1, 2, 4%), Huang and Bohrer [45] reported that, as flour inclusion increased, more water was trapped by hydrocolloids and was less available to react with proteins and less capable of maintaining elasticity, therefore springiness decreased. The chewiness was similar in all high-fat sausages during the storage period except for the HF-PUBF lot (*p* <0.05), which showed higher values during the first days of storage (Figure 1). On the contrary, LF-PEBF showed lower levels of chewiness during all chilled storage (*p* < 0.05), probably due to the lower fat content and the high fiber and carbohydrate contents that could make this batch less chewy. Regarding adhesiveness, the initial values of the Control and HF-PUBF were higher than those of the rest of the formulations (*p* < 0.05) but later they declined, while in the rest of the lots the values remained stable. Some studies on fat replacement with different kinds of flour or fiber have been reported in the literature, but it is difficult to compare their effects on the formulations considering the great variability and differences in the fat substitutes used (type and quantity), as well as in the possible variation of water or protein contents in the formulations. In short, in the present work, the substitution of fat with any other ingredient or modification in the formula is intended to change the texture parameters as little as possible to resemble the traditional product (Control). The water immobilized by the hydrocolloids and the water available to react with meat proteins during the emulsification process should be balanced [45] as it can influence the food system. In this sense, textural parameters were not affected in hot-dog type sausages in which fat was replaced in a range of 50–100% with pork skin, bamboo fiber, canola oil, and inulin emulsions [46]. Textural changes were not found in Bologna-type sausages in which up to 60% of fat was substituted with a gel formulated with pork skin, water, and banana flour in a ratio of 1:2:2 [1]. However, Pereira et al. [47] observed that the incorporation of pre-emulsified sunflower oil and green banana flour (as partial replacers of pork back fat) diminished the hardness and chewiness with respect to the Control (containing pork back fat) and increased the springiness of sausages when green banana flours were added. Despite the rheological differences observed in sausages with sunflower oil and different types of green banana flour, banana skin flour had the most negative impact on the product [47].

### 3.5. Appearance and Color

**Figure 2:**
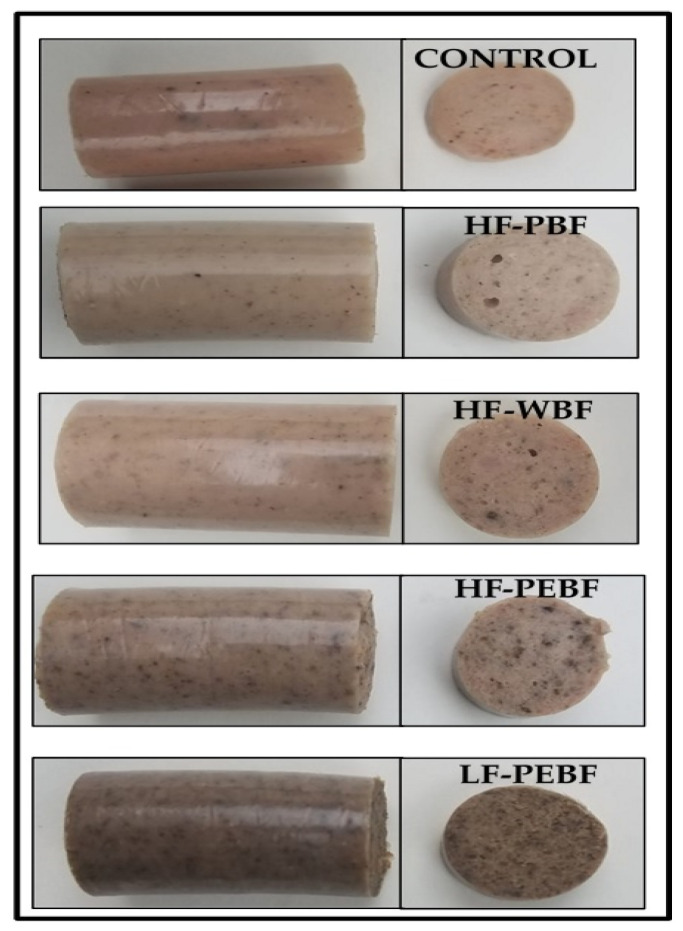
Visual appearance of Frankfurter-type sausages formulated with green banana flours: Control (High fat and wheat flour), HF-PUBF (High fat and banana pulp flour), HF-WBF (High fat and whole banana flour), HF-PEBF (High fat and banana peel flour), LF-PEBF (Low-fat and banana peel flour).

The visual appearance of the different formulations is shown in Figure 2. In this work, no coloring was added to avoid masking the appearance of the different sausages, to evaluate the effect of adding banana flours. All batches high in fat content, except those containing banana peel flour (HF-PEBF), showed a rather homogeneous aspect, similar to Frankfurter-type sausages, with slight color differences. Thus, the presence of banana peel flour increased darkness considerably, being noticeable the appearance of spots, typical of fiber presence, and which visually can be mistaken for spices, resembling sausages such as Bratwurst or even Blutwurst, when banana flour only comprises the peel and is added to the formulation in higher concentrations (LF-PEBF).

**Figure 3:**
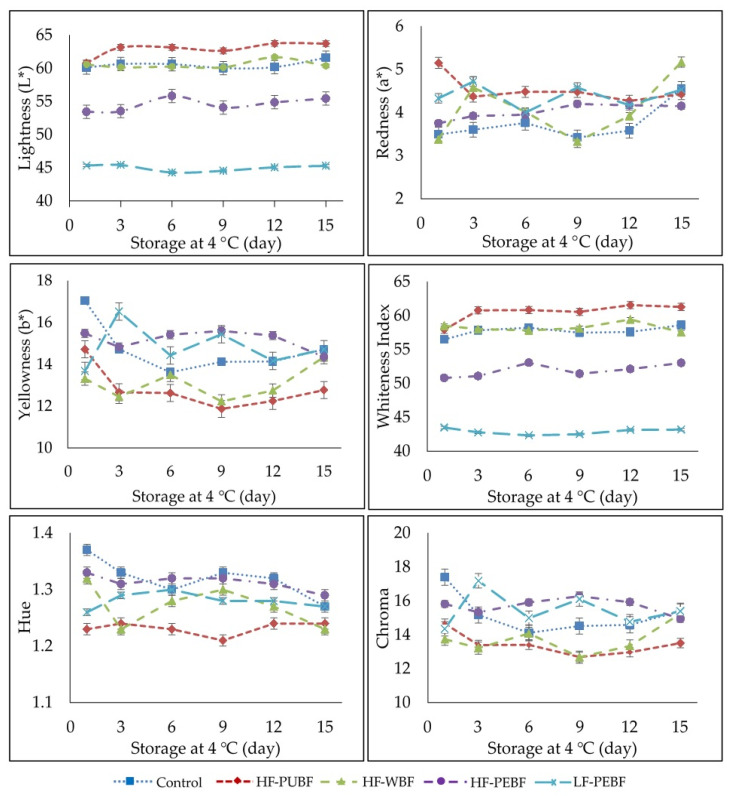
Influence of Frankfurter-type sausages color formulated with green banana flours: Control (High fat and wheat flour), HF-PUBF (High fat and banana pulp flour), HF-WBF (High fat and whole banana flour), HF-PEBF (High fat and banana peel flour), LF-PEBF (Low-fat and peel banana flour).

The influence of green banana flour in the color of Frankfurter-type sausages is shown in Figure 3. The color remained stable in all batches during storage with some differences related to their composition. Lightness (L*) was similar for the Control, HF-PUBF, and HF-WBF. During the first days of chilled storage, lightness increased in HF-PUBF (*p* < 0.05). However, it declined with the incorporation of banana peel flour, being more pronounced in the reduced-fat formulation (LF-PEBF), where the sausages acquired a darker color.

Initially, redness (a*) levels were similar for the Control, HF-PEBF, and HF-WBF (*p* < 0.05). Intermediate values corresponded to the batch with the lowest fat content (LF-PEBF), while the greatest values for redness were achieved for HF-PUBF due to the presence of banana pulp, which may show some Maillard reaction pigmentation produced during the drying process. Throughout storage, redness exhibited some fluctuations with intermediate values, however, at the end of storage similar values between 4.5–5 were reached in all batches.

Regarding yellowness (b*), the Control showed the most pronounced initial values, while the HF-WBF and LF-PEBF exhibited the lowest (*p* < 0.05). During chilled storage, all lots suffered slight oscillations, although there were two clear tendencies, LF-PEBF and HF-PEBF showed higher values, while HF-PUBF and HF-WBF presented the lowest, and the Control showed intermediate values; except for the last day when the values of almost all lots were the same (*p* < 0.05). This implies that banana peel is the main contributor to yellowness, while the presence of pulp flour favors its decrease.

Whiteness showed the same behavior as lightness, but with lower values, due to the influence of a* and b* parameters on its calculation. Accordingly, the LF-PEBF lots showed noticeably less lightness and whiteness than the other formulations.

Chroma and hue in general showed a quite stable tendency despite slight oscillations during the period studied (*p* < 0.05). The most notable were the lower hue and chroma in HF-PUBF, chroma being also low in HF-WBF.

The diverse coloration among the different batches may be due to the variation in the color of the flours and, therefore, in the mass formed by the emulsified muscle protein. The low-fat content in LF-PEBF sausages resulted in darker, intermediate red and yellow products. High-fat sausages contain a large amount of pork back fat, thus, a higher concentration of carotenoid pigments can favor the intensity of a* [48], although this trend is not clearly observed in this work. Nevertheless, the low-fat product (LF-PEBF) can alternatively obtain coloration from the banana peel flour content as well. In this sense, a significant increase in yellowness was observed in meat emulsions where fat was replaced with bran fiber [49]. As for the results obtained by other authors, variable effects have been found in chroma when fat is replaced with another ingredient in meat products. So Dzudie et al. [50], when replacing fat in meat sausages with bean/bean flour, did not observe differences, nor did Mansour and Khalil [51] when substituting fat with wheat fiber in hamburgers, while chroma values increased in cooked meat gels when adding rice bran fiber [52]. Henning et al. [25] reported that chroma decreased during the storage of sausages formulated with commercial pineapple dietary fiber, probably due to the oxidation of myoglobin to metamyoglobin, which would reduce the a* parameter, resulting in grayish-colored sausages.

### 3.6. Microbiological Analysis

**Figure 4:**
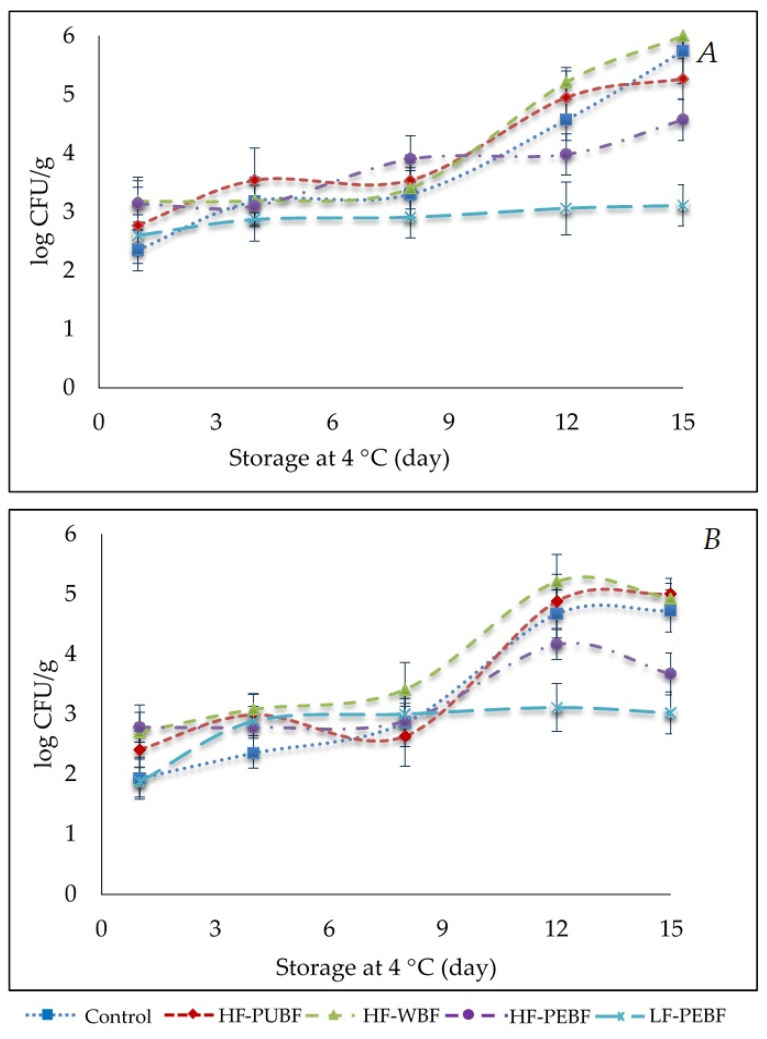
(**A**) Total aerobic mesophiles bacteria [cfu/g] and (**B**) mold and yeast [cfu/g] during chilled storage of Frankfurter-type sausages formulated with green banana flours: Control (High fat and wheat flour), HF-PUBF (High fat and banana pulp flour), HF-WBF (High fat and whole banana flour), HF-PEBF (High fat and banana peel flour), LF-PEBF (Low-fat and peel banana flour). Results are the mean ± standard deviation.

Initially, the counts of mesophilic aerobic microorganisms were 2.4–3 log CFU/g for all lots (Figure 4A). Similar results were found by Ranucci et al. [53] in pork sausages with emmer wheat, hazelnut, and almond, which reported 3.05 log CFU/g. All lots evolved similarly until day 8 of storage. From this point onward, the high fat and Control formulations showed a progressive increase (*p* < 0.05) until reaching around 5.4 log CFU/g C (C, HF-WBF, and HF-PUBF), counts being significantly lower in HF-PEBF (3.96 CFU/g) at the end of the studied period. Counts for the low-fat sausages (LF-PEBF) showed no significant changes during storage (3.14 CFU/g). There are few studies of preservation in cooked sausages using flours as fat replacers, and the formulations are so different that it is not easy to compare results. Despite this, it is worth noting that similar results were observed in other meat products. So Bologna-type sausages with 2% citrus fiber reached 5 CFU/g for aerobic bacteria after 15 days of storage [34], and pork sausages with wheat, hazelnut, and almond integrated with a mix of *Punica granatum* and *Citrus* spp. ranged from 3.8–5.19 log CFU/g (depending on the formulation) after 2 weeks of storage [53]. Cerón-Guevara et al. [54] observed higher total viable counts (among 4.52–6.12 CFU/g) in low-fat Frankfurter-type sausages with mushroom flours (2.5 and 5%), pasteurized and vacuum packed, which they attributed to spore-forming bacteria inherently present in agricultural products.

Mold and yeast counts were evaluated due to the inclusion of different quantities of flour in the formulations. A similar pattern to that described previously for total mesophilic aerobic counts was observed (Figure 4B), with LF-PEBF registering the lowest counts and to a lesser extent, HF-PEBF (*p* < 0.05). Meat product quality could be affected by the inclusion of flour -and its starch content- due to the presence and/or increment of mold and yeasts [55], given that these microorganisms use starch as a growth substrate. However, this is not in agreement with the results of the present study, taking into account that LF-PEBF presented the highest flour and starch contents. It could be possible that banana peel contains more resistant starch, but this has not been determined in the present work. However, the high content of resistant starch of this type of green banana is known [56].

Regarding *Enterobacteria* and *Staphylococcus aureus*, no counts were obtained during chilled storage in any lots. Heat treatment, as well as the addition of nitrites into the formulation, inhibits the growth of these microorganisms due to a halt in active transport, oxygen absorption, and oxidative phosphorylation in bacteria such as *Escherichia coli*, *S. aureus*, and *Clostridium botulinum* [57]. The high stability of LF-PEBF during chilled storage and, to a lower extent that of HF-PEBF, could be due to the greater fiber content, since dripping was not observed in any lot during storage. Therefore, it could be considered that water might be trapped by the fiber and therefore, less available to microorganisms.

### 3.7. Sensory Analysis

**Figure 5:**
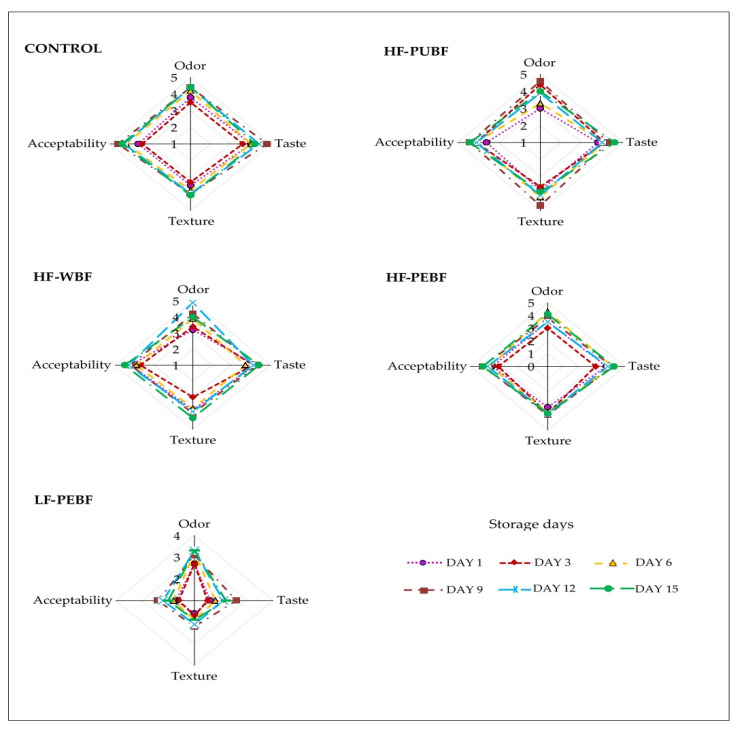
Sensorial attributes of Frankfurter-type sausages formulated with green banana flours during chilled storage. Control (High fat and wheat flour), HF-PUBF (High fat and banana pulp flour), HF-WBF (High fat and whole banana flour), HF-PEBF (High fat and banana peel flour), LF-PEBF (Low-fat and banana peel flour).

The incorporation of green banana flours hardly changed the sensory attributes of high-fat sausages (Figure 5). Control, HF-PUBF, and HF-WBF batches registered attributes with an average score of ~4 (*p* < 0.05), occasionally differing during storage by less than 0.5 points, which can be considered as negligible. Moreover, a slight increase in attribute scores with respect to the Control was observed in the high-fat sausages during this period; as in HF-PUBF for odor, taste, and acceptability and HF-WBF for odor (*p* < 0.05). The main difference found among the high-fat sausages was odor, increasing the score for this attribute during storage. No off-flavors were detected in any of the sausages during the studied period. Regarding the low-fat and flour-enriched sausages, sensory attributes were lower. Low-fat sausages were described to have an uncharacteristic smell and taste, a less pleasant texture, and therefore, the overall acceptability was lower. Regarding texture acceptability, the lower values may be due to the high hardness and low chewiness values in this sample that were obtained by mechanical methods, as previously described in Figure 1. It is noteworthy that the scores tended to increase slightly during storage (*p* < 0.05), which may indicate that the judges became more accustomed to the product with time; in fact, judges perceived these sausages as a different product. This result is in accordance with Arildsen Jakobsen et al. [58], who noticed that in fresh sausages rich in the rye (2.4%) or wheat fiber (3.2%) and 10% of fat, an increase in firmness altered the texture of the sausages, which became grainy, decreasing scores in flavor, odor, and acceptability. Similar results have also been observed, with scores around the limit of acceptability for most of the attributes in pork sausages when substituting 30 and 50% of pork fat with flours from two varieties of mushrooms in proportions of 2.5 or 5%, in which a characteristic mushroom odor was appreciated [54]. As the authors suggested, the acceptability of the product depends on how habitual the consumer is mushroom flavor. The substitution of 50% pork fat with an emulsion prepared with pork skin, canola oil, bamboo fiber, and inulin in hot-dog type sausages [46], or the substitution of up to 60% pork fat with a gel of pork skin, green banana flour, and water (in a ratio of 1:2:2) in Bologna-type sausages [1], leads to lower sensory attributes as odor, texture, and acceptability.

## 4. Conclusions

The use of banana flours (pulp, peel, or whole banana) is an ideal alternative to wheat flour to produce Frankfurt-type sausages since they have physicochemical and composition characteristics that do not differ substantially among them, in their preservation or their sensory properties. This would allow new applications at an industrial level, favoring the creation of added value products from this underused material in certain countries, with good nutritional properties and low allergenicity, gluten-free. These new Frankfurt-type products could be labeled as a “source of fiber,” except for HF-PEBF, which could bear the “high fiber” claim.

The low-fat and high-fiber sausages are different products in composition and physicochemical properties. These new sausages present higher values for hardness and a darker color, however, they preserve microbial growth-bacteria and molds and yeasts; with sensory properties in the acceptance limit, yet increasing acceptability with time when sausage awareness increases, as observed in tests performed during storage. So low-fat sausages formulated with banana flours may be candidates for consumption, especially if they are introduced as a new product, which can be labeled as “high in fiber” according to nutritional claims.

The results obtained in this research allow establishing the viability of using banana flours as a meat extender in Frankfurter-type sausages, conferring good nutritional and healthy properties.

## Figures and Tables

**Table 1:** Proximate composition, energy values, pH, and acidity (g/100 g of lactic acid), of Frankfurter-type sausages: Control (High fat and wheat flour), HF-PUBF (high fat and banana pulp flour), HF-WBF (high fat and whole banana flour), HF-PEBF (High-fat banana peel flour), and LF-PEBF (Low-fat and banana peel flour).

Properties	Control	HF-PUBF	HF-WBF	HF-PEBF	LF-PEBF
Cooking loss (%)	5.35 ± 0.05 ^b^	4.88 ± 0.03 ^d^	5.03 ± 0.03 ^c^	4.41 ± 0.04 ^e^	5.65 ± 0.05 ^a^
Moisture (%)	56.77 ± 0.12 ^c^	57.22 ± 0.14 ^b^	56.86 ± 0.14 ^c^	58.69 ± 0.04 ^a^	55.12 ± 0.07 ^d^
Ash (%)	0.63 ± 0.12 ^c^	0.22 ± 0.07 ^d^	0.44 ± 0.17 ^cd^	0.79 ± 0.21 ^b^	1.61 ± 0.07 ^a^
Protein (%)	10.15 ± 0.01 ^c^	10.49 ± 0.02 ^b^	10.71 ± 0.01 ^a^	10.50 ± 0.01 ^b^	9.56 ± 0.01 ^d^
Fat (%)	24.78 ± 0.01 ^b^	22.25 ± 0.05 ^a^	23.62 ± 0.01 ^c^	21.06 ± 0.02 ^d^	12.69 ± 0.01 ^e^
Fiber (%)	4.31 ± 0.01 ^e^	5.37 ± 0.01 ^c^	5.19 ± 0.01 ^d^	7.30 ± 0.01 ^b^	10.42 ± 0.01 ^a^
Carbohydrates (%)	3.36 ± 0.07 ^b^	4.36 ± 0.07 ^d^	3.19 ± 0.29 ^b^	1.64 ± 0.21 ^c^	10.60 ± 0.14 ^a^
Calories (Kcal/100 g)	285.66 ± 0.20 ^b^	271.2 ± 0.42 ^a^	278.53 ± 1.06 ^c^	251.67 ± 0.78 ^d^	215.71 ± 0.53 ^e^
Fat Calories (Kcal/100 g)	223.02 ± 0.05 ^a^	201.06 ± 0.14 ^c^	212.58 ± 0.10 ^b^	189.63 ± 0.18 ^d^	114.21 ± 0.14 ^e^
CH & F Calories (Kcal/100 g)	22.14 ± 0.3 ^cd^	28.18 ± 0.27 ^b^	22.64 ± 0.18 ^c^	20.08 ± 0.28 ^d^	63.22 ± 0.50 ^a^
Protein Calories (Kcal/100 g)	40.6 ± 0.05 ^c^	41.96 ± 0.06 ^b^	42.84 ± 0.04 ^a^	42.0 ± 0.05 ^b^	38.24 ± 0.02 ^d^
pH	6.69 ± 0.03 ^a^	6.56 ± 0.04 ^b^	6.54 ± 0.04 ^b^	6.45 ± 0.02 ^c^	6.42 ± 0.03 ^c^
Acidity (g/100 g)	0.63 ± 0.01 ^c^	0.75 ± 0.01 ^a^	0.62 ± 0.01 ^c^	0.63 ± 0.01 ^c^	0.69 ± 0.01 ^b^

Results are the mean ± standard deviation. One-way ANOVA: different letters (a, b, …) in the same line indicate significant differences among samples (*p* ≤ 0.05). CH: carbohydrate, F: fiber, with the energetic contribution of each one.
